# An Internet-Based Parent Training With Telephone Coaching on Managing Disruptive Behavior in Children at Special Family Counseling Centers During the COVID-19 Pandemic: Feasibility Study

**DOI:** 10.2196/40614

**Published:** 2022-11-02

**Authors:** Saana Sourander, Andre Sourander, Susanna Hinkka-Yli-Salomäki, Terja Ristkari, Marjo Kurki

**Affiliations:** 1 Department of Child Psychiatry University of Turku Turku Finland; 2 Unit of Digital Education and Master Programmes Laurea University of Applied Sciences Vantaa Finland; 3 INVEST Research Flagship University of Turku Turku Finland; 4 Department of Child Psychiatry Turku University Hospital Turku Finland; 5 ITLA Children´s Foundation Helsinki Finland

**Keywords:** parent training, disruptive behavior, child psychopathology, child functioning, internet-based, COVID-19 pandemic, COVID-19, mental health, psychological well-being, digital health, parenting, telehealth, behavioral problem, psychopathology

## Abstract

**Background:**

There is growing concern about the short- and long-term impacts that the COVID-19 pandemic will have on the mental health and psychosocial well-being of children and families. There are no existing studies about feasibility and outcomes using internet-based parent training programs with telephone coaching for disruptive behavioral problems in childhood during the COVID-19 pandemic in clinical settings.

**Objective:**

This study explored how the Strongest Families Smart Website (SFSW) parent training program, with telephone coaching, provided support during the COVID-19 pandemic at specialist family counseling centers in Helsinki, Finland, when restrictions made face-to-face counseling impossible. This study followed the success of a randomized controlled trial (RCT) and its implementation study of the SFSW parent training program by primary care child health clinics. The aim was to improve parenting skills, so that parents could tackle disruptive behavior by developing positive parent-child relationships. It started in May 2020, when the COVID-19 pandemic was at its height in Finland.

**Methods:**

In total, 8 family counseling centers in Helsinki identified 50 referrals aged 3-8 years with high levels of parent-reported disruptive behavioral problems. Child psychopathology and functioning and parental skills and well-being were measured at baseline, posttreatment, and 6 months later using a range of tools. The data were extracted from questionnaires completed by the parents.

**Results:**

We found that 44 (88%) of the 50 families completed the whole 11-session parent training program. Most of the children (n=48, 96%) had definitive or severe behavioral problems when they were initially screened by the centers, but with those assessed at the 6-month follow-up (n=45, 90%), this dropped to 58% (n=26). There were significant changes from baseline to 6-month follow-up in most of the child psychopathology measures, including the Child Behavior Checklist-Parent Report Form (CBCL) total score (mean change 16.3, SE 3.0, 95% CI 10.2-22.3; *P*<.001) and externalizing score (mean change 7.0, SE 1.0, 95% CI 4.9-9.0; *P*<.001). When parenting skills were measured with the Parenting Scale (PS), they showed significant changes from baseline to 6-month follow-up in total scores (mean change 0.5, SE 0.1, 95% CI 0.4-0.7; *P*<.001). Parents showed significant change in the stress subscore (mean change 3.9, SE 0.8, 95% CI 2.2-5.6; *P*<.001). Of the parents who filled in the satisfaction questionnaire (n=45, 90%), 42 (93%) reported high satisfaction in the skills and 44 (98%) in the professionalism of the family coaches.

**Conclusions:**

The program proved to be an effective method for improving parenting skills and child psychopathology and functioning. The parents were satisfied with the program, and the dropout rate was exceptionally low. The study shows that the training program could be implemented in specialist clinical settings and during crisis conditions, such as the COVID-19 pandemic.

## Introduction

There is growing concern about the possible short- and long-term impacts that the COVID-19 pandemic is having on the mental health and psychosocial well-being of children and their families [1]. Studies have shown that the use of mental health services by children and adolescents was lower during the initial phase of the COVID-19 pandemic than before the pandemic [2]. However, some time-trend studies have shown that mental health problems have increased during the COVID-19 pandemic [3-5]. This has resulted in a higher level of unmet needs in children with mental health problems. These findings have underlined the need for low-threshold and remote services to address the psychosocial problems affecting children and their families. It is crucial that we be able to demonstrate the feasibility and outcomes of such programs in real-world settings during the COVID-19 pandemic because they are likely to prove invaluable during both current and future crises.

Disruptive behavior and conduct problems are common among children and can lead to negative outcomes in later life [6-9]. Children with disruptive behavior and conduct problems have higher risks of encountering lifelong disorders in relation to conduct, impulse control, mood, anxiety, suicidality, and substance abuse [7-10]. It is likely that several risk factors linked to the COVID-19 pandemic will have detrimental effects on children, and these are particularly expected to affect vulnerable children, such as those with disruptive behavior problems. These risk factors could include isolation due to school closures, parental stress about the virus and job security, increases in undetected child abuse, greater levels of cyberbullying due to increased online activities, and the trauma or threat of losing family members [1,11-13].

Parent training has been found to be the most effective way to prevent and treat disruptive behavioral problems among children. There is growing evidence from randomized controlled trials (RCTs) that such initiatives reduce problems and improve parenting skills [14-17]. Parent training has been shown to be 1 of the best-validated therapeutic techniques in child mental health [18]. Interventions that encourage positive behavior, and include video demonstrations, practical exercises, and homework, have helped parents reduce their children’s aggressive behavior. The goal of these interventions is to teach parents to identify, define, and observe their children’s problem behaviors in new ways. They also teach parents strategies that help them prevent their child’s oppositional behavior and react to any episodes in a positive way [15]. Parent training should be the first choice when it comes to tackling children’s disruptive behavior [19]. Despite this, only a small percentage of families who are struggling with these problems receive evidence-based treatment programs [20]. The biggest barriers to such programs include the stigma related to receiving mental health treatment and the difficulties in accessing, and engaging with, treatment programs, because of time, cost, and location [16,17]. Providing traditional parent training programs has been challenging during the COVID-19 pandemic restrictions, particularly as they are usually based on group treatments and face-to-face contact. Problems have been exacerbated by lockdowns and other social distancing measures, together with fears of getting infected by the virus during face-to-face contact and a possible decrease in seeking help when problems arise. One consequence of the COVID-19 pandemic could be the decreased availability of evidence-based parent training interventions for children with disruptive behavior. Delaying these interventions, or not being able to provide them, could lead to further deterioration in the children’s problems and functioning levels. There are also concerns that steps taken to impede the spread of the pandemic may have also led to increased risk family dysfunction, which may have had a particular impact on vulnerable children, including those with disruptive behavior problems [21,22].

A number of studies have found that many digital and digital-assisted parent training programs offer many benefits over traditional interventions, such as high levels of support, higher fidelity, greater accessibility, and convenience [23-26]. They can also reduce health care costs and time.

Our pioneering Strongest Families Smart Website (SFSW) study was the first RCT to use an internet-based intervention, with telephone coaching, to train the parents of Finnish preschool children with disruptive behavior [27]. They were identified by public health nurses at routine 4-year child health clinic health check-up visits [28]. The 11-week internet-based parent training intervention comprises parent training material delivered via an interactive online platform, which is backed up by regular telephone contact with specially trained coaches. This intervention has been shown to improve the preschool children’s psychiatric symptoms and the parents’ skills in handling their disruptive behavior. The RCT showed that improvements were maintained 24 months after the program, when the families who received the intervention were compared with a control group that only received basic information on the subject [29-32].

This paper is the first to report the feasibility and outcomes of providing the SFSW program in a clinical setting during the COVID-19 pandemic. The first aim was to report changes in the children’s functioning and psychopathology levels at baseline, posttreatment, and 6 months after baseline. The second aim was to report changes in parenting skills and parent well-being at the same time points. The third aim was to shed light on the feasibility of providing an internet-based training program in specialist clinical settings during exceptional circumstances, namely family counseling centers and the COVID-19 pandemic. Based on using the SFSW in primary health care settings, we hypothesized that the parent training program could show significant reductions in a wide range of child psychopathology problems, increase parenting skills, and reduce parental stress. We also expected a high satisfaction level and a low dropout level during the program.

## Methods

### Study Environment

The study focused on clients from each of the 8 family counseling centers in Helsinki, the capital of Finland, where social workers, psychologists, and doctors offer low-threshold services that are based on openness and confidentiality. The centers are administratively part of social services and support the child’s development by strengthening parenting skills and relationships between the child, parents, and other family members. Families can themselves contact the centers, or they can be referred by child health centers or other health care professionals. The centers work as part of a network with other organizations, such as schools, social services, and child protection. This means that families benefit from multiprofessional support that is integrated into any other support plans they have.

The family counseling centers provide specialist support for children and adolescents aged 0-17 years when basic services are not enough for them and their family. Direct support is offered at the centers, and center staff can also provide advice to other services who are helping the families. The centers can also refer children and families to other specialist services, such as child protection and child psychiatry.

Family counseling centers typically offer parent training as individual face-to-face meetings or in group sessions, and these cover areas such as problems raising children or crisis situations. During the COVID-19 pandemic, there were lockdowns and these face-to-face services were impossible to arrange. It was not possible to offer face-to-face or group-based guidance, and this highlighted the importance of providing parental support in other ways, including our SFSW internet-based parent training program with telephone coaching.

### Study Design

This study had a single-group design with repeated measurements. The parents were asked to fill in questionnaires at baseline, posttreatment, and 6 months after starting the parent training program. The baseline questionnaires were filled in before the program started, the posttreatment questionnaires right after the program ended, and the baseline questionnaires 6 months later. The study population comprised 50 families. The study was conducted between May 2020 and September 2021. When the study started, the COVID-19 pandemic situation was at its height in Helsinki and a state of emergency had been declared across Finland. There were strict social distancing restrictions in the Helsinki area to try to halt the spread of the virus, and these had a big impact on families living in the area. Schools and leisure facilities were closed, social contact was strictly limited, and most parents who were able to work from home did so.

### Study Population

This study focused on children aged 3-8 years who displayed high levels of disruptive behavior when they were screened by 8 family counseling centers. The study population comprised 50 families, and 37 (74%) of the 50 children aged 3-8 years were boys. Staff from the 8 counseling centers identified the families they felt would benefit from the SFSW internet-based parent training program, with telephone coaching, for children with disruptive behavioral problems.

### Recruitment

The screening measures and enrollment criteria were identical for the implementation study carried out at the counseling centers, the previous child health care clinic implementation study, and the original RCT [29-32]. The screening was mainly carried out using the conduct scale of the Strengths and Difficulties Questionnaire (SDQ) [33,34]. Parents who were already attending the counseling center before the pandemic started were asked whether their child had mild, moderate, or severe problems. This was based on a single question about whether the child had difficulties in 1 or more of the following areas: emotions, behavior, or getting on with other people. If they replied yes, then they met the first inclusion criterion. They were also asked whether they felt that their child had at least minor difficulties when it came to emotions, behavior, or social interactions. To take part in the study, at least 1 parent had to speak native Finnish or Swedish and they needed access to a telephone and a device with an internet connection. The exclusion criteria included children who had been diagnosed with autism; Down syndrome; fetal alcohol syndrome; an intellectual disability; a severe mental disorder, such as psychosis or depression; or genetic-based mental retardation. We also excluded children who were unable to speak, had difficult hearing, or had visual impairments that were not corrected by wearing glasses.

### Procedure

Families were approached about the study if they met the eligibility criteria and would derive the most benefit from the SFSW parent training program by clinical evaluation at the family counseling center. The whole parent training program and data collection were carried out from 1 center, the Research Centre for Child Psychiatry, University of Turku, Finland. If parents agreed, they were provided with password-protected access to the internet site and allocated a family coach for the duration of the program. They started the program by completing a series of questionnaires (at baseline) and then worked through the 11 sessions, with weekly guidance from the family coach (Table 1). When they completed the program, they were asked to fill up the posttreatment questionnaires and provide feedback on the program. The data collected at baseline were compared with the data collected after the program and 6 months after the program started to measure the impact of the program on the parents and the children.

**Table 1:** Themes of the 11-session SFSW^a^ internet-based parent training program for children with behavioral problems.

Session	Key training elements	Goals
1. Notice the good.	Positive, active parenting	Boost the child’s self-esteem, boost the parent’s self-esteem, and change the parent’s view of their child.
2. Spread attention around.	Positive, impartial parenting	Strengthen the child’s empathy skills.
3. Ignore whining and complaining.	Positive, self-controlled parenting	Teach parents self-regulation.
4. Prepare for changes.	Positive, proactive parenting	Reinforce good daily routines.
5. Plan ahead at home.	Positive, proactive parenting	Boost the self-esteem of the child and the parent and involve the child in planning.
6. Chart and stickers.	Positive, active parenting	Involve the child in planning and reinforce good daily routines.
7. Plan ahead outside the home.	Positive, proactive parenting	Boost the self-esteem of the child and the parent and involve the child in planning.
8. Working with day care.	Positive cooperation and communication between parent and day care	Help the child manage and succeed.
9. Time out.	Positive, self-controlled parenting	Teach self-regulation and consistency.
10. and 11. Revise problem solving and future application of skills.	Positive daily parenting in the future	Teach parents skills to support child development and prepare for future challenges.

^a^SFSW: Strongest Families Smart Website.

### Intervention

The intervention was originally developed from the Canadian version of the Strongest Families intervention, which was provided through handbooks, videos, and weekly telephone calls from the coach [35]. In our study, the participants received the intervention, which was the internet-based SFSW parent training program. The SFSW parent training program comprised material delivered via an interactive online platform and telephone coaching. Although it was based on 11 weekly themes, some parents needed longer to progress to each new stage. The program focused on improving skills to strengthen parent-child relationships, together with a series of weekly telephone sessions with specially trained coaches. The family coaches were licensed health care professionals, such as nurses and public health nurses. Each family coach received a training for the internet-based program held by experienced coach supervisors. The training included theoretical information (eg, mental health prevention methods and information about conduct problems in childhood) and rehearsal phone calls [31]. After receiving the training, the family coach was ready to start carrying out the program with the families.

All the coaching calls were recorded, and the recorded calls were audited by the coach supervisor randomly. After each coaching call, the family coach assessed their own performance on a scale from 4 to 10. If self-assessment was equal to 6 or less, the coach supervisor received a message from the digital platform and subsequently discussed the issue with the family coach. There were also systematic supervision meetings with each family coach, if needed, and weekly group case meetings, where all family coaches reviewed and discussed the families they were coaching [31]. A rough estimate of the direct costs, including coaching, supervision, IT support, contacts with the family counseling centers, and administrative, postage, and material costs, were approximately €1500 (US $ 1468.42) per family.

The program started by discussing and on agreeing personalized goals for the program based on the child’s behavior problems. The sessions were divided into 3 sections: basic positive parenting skills, practical parenting skills and reinforcing the skills they had acquired, and sustaining their approach to positive parenting. During the first 7 weeks, parents learned positive and practical problem-solving skills and were encouraged to develop an understanding of their child’s emotional development.

The primary aim was that the parent would notice the child’s positive behavior and react with a positive response. The second aim was to apply the skills they had learned in everyday situations and use positive methods to reinforce the child’s positive behavior. The last 2 weekly themes focused on reinforcing the use of their new positive parenting skills in everyday life in order to support their child’s positive behavior. The parents practiced their positive parenting skills with their child and discussed their progress during the weekly telephone calls with their coach. The goal was to ensure that the parents were able to sustain the skills they had learned when the program finished. The weekly themes are depicted in Table 1.

### Measurements

The parents completed online questionnaires at baseline, after the parent training program, and 6 months after they had started the program. The timing of each questionnaire is described in Table S1 in Multimedia Appendix 1.

### Demographic and Family Information

Demographic information was obtained at the screening phase and included the child’s sex, the family structure, and the parents’ birth year, native language, educational level, and employment status. The demographic and family information are depicted in the Results section.

### Child Psychopathology and Functioning

Psychopathology was measured using the SDQ [33,34], a brief behavioral screening questionnaire that examines positive and negative behaviors in subjects aged 3-16 years. The 25 items of the SDQ are divided into 5 subscales of 5 questions: emotional symptoms, conduct problems, hyperactivity/inattention, peer relationship problems, and prosocial behavior. Perceived difficulties were assessed with a single question about whether the child had difficulties in at least 1 of these areas: emotions, behavior, or being able to get on with other people. The possible answers were no, minor difficulties, definite difficulties, and severe difficulties. One study reported that the SDQ had an internal consistency score of 0.58 when it was used by the parents of preschool children [36].

Child irritability was measured by the Affective Reactivity Index (ARI) scale, which comprises 6 irritability symptom items and 1 impairment item [37]. The ARI scale examines 3 aspects of irritability: the threshold for an angry reaction, the frequency of angry feelings/behaviors, and the duration of such behaviors/feelings. Parents were asked to assess their child’s behavior over the past 6 months compared to peers of the same age. They were presented with 6 statements about behaviors and feelings related to irritability and were asked to say whether they were not true (0 points), somewhat true (1 point), or certainly true (2 points). The ARI scale also includes 1 question about whether the child’s irritability impairs them, with the same possible responses.

Disruptive behavior was measured by the externalizing subscale of the Child Behavior Checklist-Parent Report Form (CBCL) for ages 1.5-5 years. The CBCL 1.5-5 [38] comprises 99 problem items, and the subscales are emotionally reactive, anxious/depressed, somatic complaints, withdrawn, sleep problems, attention problems, and aggressive behavior. These can be combined to provide internalizing, externalizing, and total problem scores. This study focused on the externalizing subscale, which comprises 24 items on behavioral problems, including attention issues and aggressive behavior, and the total score of the CBCL. The parents were asked to evaluate their child’s behavior during the past 2 months using a 3-point scale for each item: 0 (not true), 1 (somewhat true), and 2 (very true/often true). The CBCL has good test-retest reliability (eg, 0.81) and criterion validity (eg, 0.56-0.87) [38].

The 24-item Inventory of Callous-Unemotional Traits (ICU) [39] is used to evaluate 3 precursors of psychopathy: callousness, uncaring, and unemotional traits. It has been shown to be an important measure for identifying subgroups of antisocial and aggressive children and adolescents [40,41]. The ICU comprises 24 statements with a 4-point Likert scale: 0 (not at all true), 1 (somewhat true), 2 (very true), and 3 (definitely true). Larger scores indicate higher callous and emotional traits.

A 17-item questionnaire, based on the Barkleys’ Home situation Questionnaire [42], was created to measure parents’ experiences of their child’s functioning and behavior during daily situations and routines. The questionnaire included questions about how the child behaved at home; in transition situations, such as when they were getting dressed; and while eating. The questionnaire asked parents about how their child behaved on a 5-point scale ranging from 1 point if the child’s behavior was easy to 5 points if it was awkward.

### Parenting, Parental Mental Health, and Satisfaction

The 30-item Parenting Scale (PS) is used to measure parenting and discipline styles for children aged 1-12 years, particularly those related to the development or maintenance of child disruptive behavior [43,44]. The scale focuses on 3 dysfunctional discipline styles: laxness, overreactivity, and verbosity. Laxness comprises 11 items about how parents fail to enforce rules. Overreactivity has 10 items on mistakes, such as displays of anger or irritability. Verbosity has 7 items that reflect lengthy verbal responses to situations. The 7-point scale ranges from ineffective to effective responses and is often used to evaluate parent training programs. The parents were asked to evaluate their parenting skills during the preceding 2 months.

The parents’ stress, anxiety, and depression symptoms during the past week were evaluated with the shorter 21-item Depression, Anxiety, and Stress Scale (DASS-21) [45]. The 3 DASS-21 scales contain 7 items, divided into subscales with similar content. For example, the depression scale assesses dysphoria, hopelessness, and lack of interest, and the anxiety scale assesses situational anxiety, autonomic arousal, and skeletal muscle effects. The stress scale is sensitive to levels of chronic nonspecific arousal, such as being easily upset and having difficulty relaxing. Responses are based on a 4-point Likert scale: 0 (did not apply to me at all), 1 (applied to me to some degree or some of the time), 2 (applied to me to a considerable degree or a good part of the time), and 3 (applied to me very much or most of the time).

Parents were also asked about their satisfaction with the parent training program when they completed the program. The same satisfaction questionnaire was used in our previous studies [31]. The satisfaction questionnaire included parents’ general experiences of the program, how it had affected their parenting skills, and their views on the website, the content of the program, and working with the telephone coach. The questionnaire also included questions about where they had gone through the program (eg, at home or work) and whether they had input from the other parent when they used the website. Each statement on the program was rated using a 5-point scale: completely disagree, disagree, not agree or disagree, agree, and totally agree (see Table S2 in Multimedia Appendix 2).

### Statistical Analysis

All participating families (N=50) were included in the intent-to-treat analyses. Categorical demographic variables, including child, parent, and family characteristics, are presented as numbers and percentages. Continuous demographic variables including the parents’ age are presented as means and SDs. The outcome variables were analyzed with linear mixed-effect models for repeated measurements with time as the within factor: at baseline, after the program (posttreatment), and at 6 months after starting the program. We used linear contrasts to estimate the changes from baseline to 6 months and, if feasible, from baseline to posttreatment and from posttreatment to 6 months. Statistical significance was judged at *P*<.05. The statistical analyses were performed using SAS statistical software, version 9.4 (SAS Institute Inc).

### Ethical Considerations

Ethical approval for the study was received from the University of Turku (statement 25/2018), and the study had a research permit from the city of Helsinki. The parents provided written informed consent and were advised that participation in the study was voluntary and they had the right to withdraw at any time.

## Results

### Participant Characteristics

The study comprised 50 families, and 44 (88%) completed the whole SFSW program, including the assessment after the program. In addition, 45 (90%) of the 50 families completed the assessment 6 months after baseline data were collected. The children were 3-8 years old, and 37 (74%) of the 50 children were boys. The baseline data showed that 38 (76%) of the 50 children lived with both their biological parents. Table 2 provides the demographics of the families included in this study and shows that 48 (96%) of the 50 children had definitive or severe behavioral problems at baseline. Only 2 (4%) of the 50 children had minor problems.

As shown in Table 2, the average time spent on the program website for each of the 11 themes was 48.0 (SD 25.6) minutes and the mean duration of telephone coaching was 35.3 (SD 8.8) minutes per call. The parents spent approximately 8-9 hours on the whole program. The average total time for 11-week phone coaching per family was 352.5 (SD 113.3) minutes. In addition, the family coaches spent time in reviewing the case; taking notes and possible remarks, if needed, after the calls; and writing the feedback, which was sent to the family counseling center and home to the family after the program. In some cases, family coaches had to be in contact with the family counseling centers. The estimated time spent by the family coach per family was approximately 9 hours total in completed programs.

Baseline, posttreatment, and 6-month follow-up scores of all child and parent outcome measures are presented in Tables 3-8. Table 4 shows the change in overall perceived behavior problems based on the single SDQ question about whether the child had overall problems in 1 or more of the following areas: emotions, behavior, or getting on with other people. This showed that 18 (36%) of the 50 children had severe problems and 30 (60%) of the 50 children had definite problems at baseline. At the 6-month follow-up, 5 (11%) of 45 children had severe problems and 21 (47%) of 45 children had definite problems. Only 2 (4%) of the 50 children had minor problems at baseline, and this increased to 19 (42%) of 45 children at the 6-month follow-up, which was a significant decrease in severity levels.

Additional analysis for those 45 (90%) of the 50 parents who completed the 6-month follow-up questionnaires showed that 35 (78%) of the 45 children had an SDQ total score above the 90th percentile (ie, abnormal range) at baseline, while only 12 (27%) remained in the abnormal range at the 6-month follow-up (*P*<.001, McNemar test) based on the population sample of 4-16-year-old children [33]. When using the 80th percentile cut-off point (ie, abnormal or borderline range), 42 (93%) children were above the cut-off point at baseline, while the respective figure at the 6-month follow-up was 23 (51%) children, indicating a highly significant change (*P*<.001).

**Figure 1:**
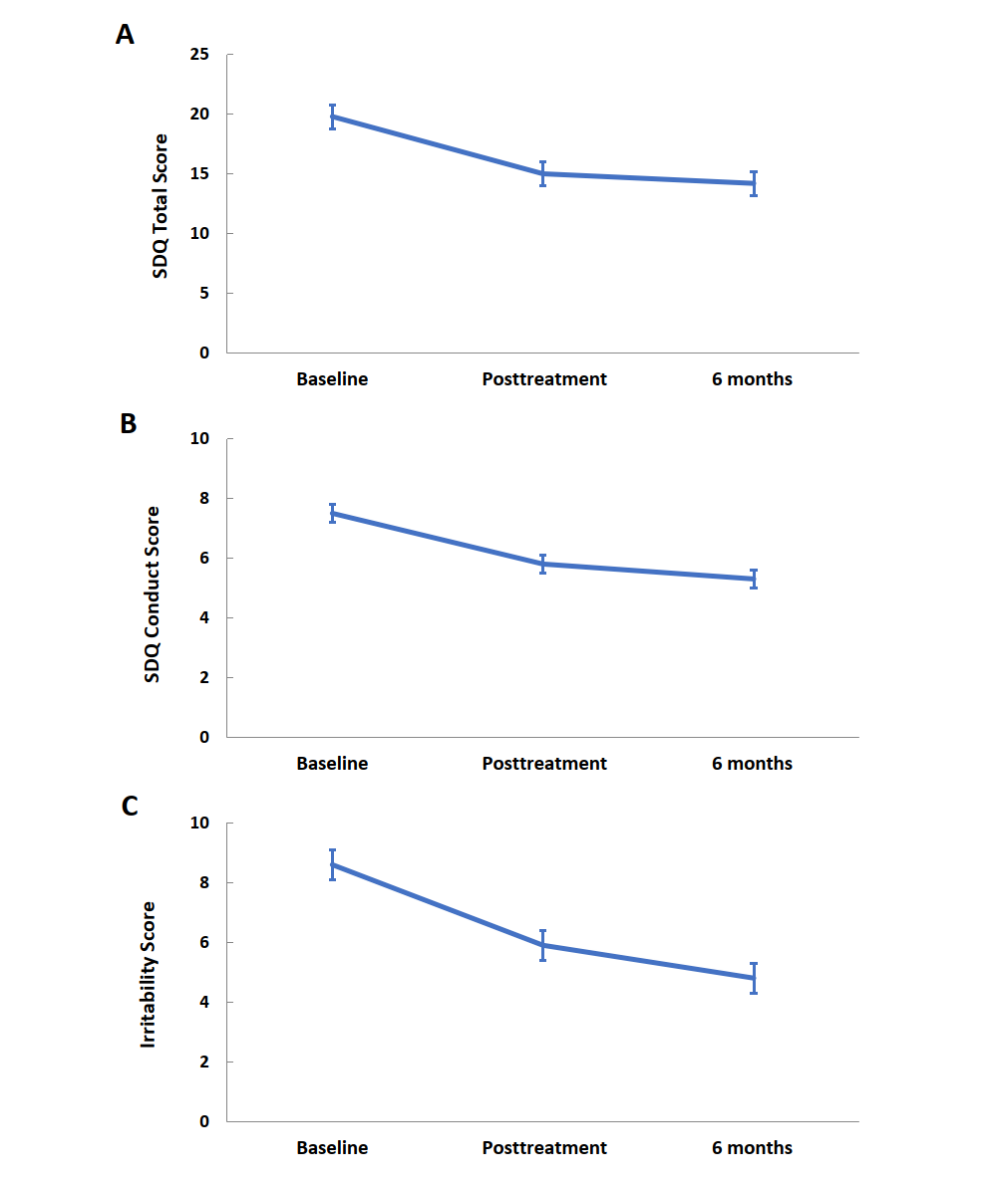
Mean curves of SDQ total and conduct scores as well as irritability score. (A) SDQ total scores over time (model-based least-squares means [SE]). (B) SDQ conduct scores over time (model-based least-squares means [SE]). (C) Irritability score over time (model-based least-squares means [SE]). SDQ: Strengths and Difficulties Questionnaire.

As shown in Table 6, there were significant improvements in most of the child psychopathology measures between baseline, before the program started, and 6 months after baseline. The only exception was the unemotional score in the ICU scale, which did not show a significant improvement. The improvements in externalizing, internalizing, hyperactivity and peer problems, irritability, and prosocial behavior measured by the SDQ, ARI, and CBCL scales were significant between baseline and 6 months. As shown in Tables 6-7, similar significant improvements were shown in the SDQ impact scale and parents’ experiences of their child’s functioning and behavior during daily situations and routines. Changes to key outcomes, namely the SDQ total, conduct, and irritability scores are visualized in Figure 1.

As shown in Table 8, when parenting skills were measured with the PS, it showed significant improvements between baseline and the 6-month follow-up. Parental mental health, which was measured with the DASS-21, showed significant improvement in the total scores and subscore measuring stress between baseline and 6 months. However, there were no significant changes in depression and anxiety.

The satisfaction questionnaire was completed by 45 (90%) of the 50 parents once they had completed the program. As shown in Table S2 in Multimedia Appendix 2, there were high levels of satisfaction with how the program had improved their parenting skills, matching their expectations and needs. More than 90% (n=42-44, 93%-98%) reported high satisfaction in the skills and professionalism of the family coaches. These findings were similar to the original RCT and child health clinic center implementation study [29-32].

Only 6 (12%) of the 50 parents failed to complete the whole program: 3 (6%) dropped out during the first few weeks, and the other 3 (6%) completed the first 7 weeks of the program, which comprise the key elements. This meant that those 3 families missed out on weeks 8-11, which focused on putting the skills and techniques they had learned into action (Table 1). In addition, 1 (17%) of these 6 families took part in the 6-month follow-up assessments. Meta-analysis shows that online parenting programs are effective in reducing children’s disruptive behavior compared to a control group and seem to have the same effectiveness as face-to-face programs [46,47]. The explaining factor for the good completion rates included highly structured and manualized content, the implementation strategy, remote delivery using phone coaching and a digitalized platform, and fidelity assurance. Special attention was given to motivate the parents to complete the program using, for example, attributional questions. To achieve good completion rates, it was important to collaborate closely with the family counseling centers.

**Table 2:** Demographic characteristics and treatment factors (N=50).

Participant and program characteristics		Participants
**Family structure, n (%)**		
	Biological parents	38 (76)
	One biological parent	11 (22)
	Other	1 (2)
**Age of the parent (years), mean (SD)**		
	Maternal	31.9 (4.3)
	Paternal	32.8 (3.7)
**Maternal educational level^a^, n (%)**		
	Secondary education	11 (22)
	College or university degree	37 (76)
	Other	1 (2)
**Paternal educational level^b^, n (%)**		
	Elementary school or less	3 (7)
	Secondary education	11 (24)
	College or university degree	31 (7)
	Other	1 (2)
**Native language of the participating parent^c^, n (%)**		
	Finnish	43 (88)
	Swedish	5 (10)
	Other	1 (2)
**Sex of the child, n (%)**		
	Female	13 (26)
	Male	37 (74)
**Age of the child (years), n (%)**		
	3-4	15 (30)
	5-6	27 (54)
	7-8	8 (16)
**Child’s behavioral problems, n (%)**		
	Minor	2 (4)
	Definite	30 (60)
	Severe	18 (36)
**Program characteristics** **, mean (SD)**		
	Mean duration of calls for the 11 themes (minutes)	35.3 (8.8)
	Mean duration of website access per theme (minutes)	48.0 (25.6)
	Total mean duration of program per theme (minutes)	83.3 (28.0)

^a^1 missing observation.

^b^4 missing observations.

^c^1 missing observation.

**Table 3:** Child psychopathology at baseline, posttreatment, and 6 months after baseline.

Variable		Baseline^a^ (N=50), mean (SE)	Posttreatment^b^ (n=44), mean (SE)	Follow-up after 6 months^c^ (n=45), mean (SE)
**SDQ^d^**				
	Total	19.8 (0.7)	15.0 (0.7)	14.2 (0.7)
	Emotional symptoms	3.5 (0.3)	1.9 (0.2)	2.2 (0.3)
	Conduct problems	7.5 (0.2)	5.8 (0.3)	5.3 (0.2)
	Hyperactivity	6.0 (0.3)	5.3 (0.3)	4.7 (0.3)
	Peer problems	2.8 (0.3)	2.1 (0.2)	2.1 (0.2)
	Prosocial behavior	5.2 (0.3)	5.6 (0.3)	6.0 (0.3)
	Impact	3.0 (0.3)	1.9 (0.3)	1.7 (0.3)
**Questionnaire for irritability**				
	Irritability	8.6 (0.4)	5.9 (0.5)	4.8 (0.4)
**CBCL^e^ for preschool children ^f^**				
	Externalizing	25.7 (1.0)	N/A^g^	18.8 (1.2)
	Total	62.1 (3.1)	N/A	45.8 (3.3)
**ICU^f,h^**				
	Total	27.4 (0.4)	N/A	23.3 (1.2)
	Callousness	8.9 (0.5)	N/A	6.8 (0.5)
	Uncaring	14.5 (0.5)	N/A	12.5 (0.6)
	Unemotional	4.1 (0.4)	N/A	4.1 (0.4)

^a^Measurements before the program started.

^b^Measurements after completing the program.

^c^Measurements 6 months after starting the program.

^d^SDQ: Strengths and Difficulties Questionnaire.

^e^CBCL: Child Behavior Checklist-Parent Report Form.

^f^The CBCL externalizing scores and total scores and the ICU were measured only at baseline and 6 months after baseline.

^g^N/A: not applicable.

^h^ICU: Inventory of Callous-Unemotional Traits.

**Table 4:** Child function level at baseline, posttreatment, and 6 months after baseline.

Variable		Baseline^a^ (N=50)	Posttreatment^b^ (n=44)	Follow-up after 6 months^c^ (n=45)	
**Everyday situations, mean (SE)**					
	Child behavior total	43.0 (1.6)	36.8 (1.4)	33.5 (1.9)	
	Transition situations	14.7 (0.6)	12.8 (0.6)	11.4 (0.6)	
	Dining situations	7.8 (0.4)	6.6 (0.3)	6.0 (0.4)	
	Situations outside home	10.4 (0.5)	8.8 (0.4)	8.1 (0.6)	
	Home situations	10.0 (0.4)	8.6 (0.4)	8.0 (0.6)	
**Behavior problems, n (%)**					
	No or minor problems	2 (4.0)	12 (27.3)	19 (42.2)	
	Definite	30 (60.0)	24 (54.5)	21 (46.7)	
	Severe	18 (36.0)	8 (18.2)	5 (11.1)	

^a^Measurements before the program started.

^b^Measurements after completing the program.

^c^Measurements 6 months after starting the program.

**Table 5:** Parental skills and parental mental health at baseline, posttreatment, and 6 months after baseline.

Variable		Baseline^a^ (N=50), mean (SE)		Posttreatment^b^ (n=44), mean (SE)	Follow-up after 6 months^c^ (n=45), mean (SE)	
**PS^d,e^**						
	Total		3.5 (0.1)	N/A^f^	2.9 (0.1)	
	Laxness		2.8 (0.1)	N/A	2.5 (0.1)	
	Overreactivity		4.3 (0.2)	N/A	3.4 (0.2)	
	Hostility		1.9 (0.1)	N/A	1.6 (0.1)	
**DASS-21^e,g^**						
	Total		22.6 (2.1)	N/A	16.8 (2.1)	
	Depression		6.6 (1.0)	N/A	4.9 (0.8)	
	Anxiety		2.8 (0.6)	N/A	2.7 (0.7)	
	Stress		13.2 (0.9)	N/A	9.3 (0.9)	

^a^Measurements before the program started.

^b^Measurements after completing the program.

^c^Measurements 6 months after starting the program.

^d^PS: Parenting Scale.

^e^The PS and DASS-21 were measured only at baseline and 6 months after baseline.

^f^N/A: not applicable.

^g^DASS-21: 21-item Depression, Anxiety, and Stress Scale.

**Table 6:** Treatment comparisons of child psychopathology at baseline, posttreatment, and 6 months after baseline.

Variable		Baseline^a^ to posttreatment^b^			Baseline to 6-month follow-up^c^			Posttreatment to 6-month follow-up	
		Mean (95% CI)	*P* value	Mean (95% CI)		*P* value	Mean (95% CI)		*P* value
**SDQ^d^**									
	Total	4.8 (3.3 to 6.2)	<.001	5.5 (4.2 to 6.9)		<.001	0.8 (–0.5 to 2.0)		.21
	Emotional	1.6 (1.0 to 2.2)	<.001	1.3 (0.7 to 1.9)		<.001	–0.3 (–0.8 to 0.2)		.21
	Conduct	1.7 (1.1 to 2.3)	<.001	2.2 (1.7 to 2.7)		<.001	0.5 (–0.1 to 1.0)		.08
	Hyperactivity	0.7 (0.1 to 1.3)	.02	1.3 (0.7 to 1.9)		<.001	0.6 (0.2 to 1.0)		.01
	Peer	0.7 (0.3 to 1.2)	.002	0.7 (0.3 to 1.1)		.001	0.0 (–0.4 to 0.4)		.99
	Prosocial	–0.5 (–1.0 to 0.1)	.08	–0.8 (–1.3 to –0.3)		.001	–0.4 (–0.7 to 0.0)		.05
	Impact	1.0 (0.5 to 1.6)	.001	1.2 (0.7 to 1.8)		<.001	0.2 (–0.3 to 0.7)		.47
**Questionnaire for irritability**									
	Irritability	2.8 (1.8 to 3.7)	<.001	3.9 (3.0 to 4.8)		<.001	1.1 (0.4 to 1.9)		.003
**CBCL^e,f^ for preschool children**									
	Externalizing	N/A^g^	N/A	7.0 (4.9 to 9.0)		<.001	N/A		N/A
	Total	N/A	N/A	16.3 (10.2 to 22.3)		<.001	N/A		N/A
**ICU^h^**									
	Total	N/A	N/A	4.1 (2.1 to 6.1)		<.001	N/A		N/A
	Callousness	N/A	N/A	2.1 (1.0 to 3.1)		<.001	N/A		N/A
	Uncaring	N/A	N/A	2.0 (1.0 to 3.0)		<.001	N/A		N/A
	Unemotional	N/A	N/A	0.1 (–0.6 to 0.7)		.88	N/A		N/A

^a^Measurement before the program started.

^b^Measurement after the program ended.

^c^Measurements 6 months after starting the program.

^d^SDQ: Strengths and Difficulties Questionnaire.

^e^CBCL: Child Behavior Checklist-Parent Report Form.

^f^The CBCL externalizing scores and total scores and the ICU were measured only at baseline and 6 months after baseline.

^g^N/A: not applicable.

^h^ICU: Inventory of Callous-Unemotional Traits.

**Table 7:** Treatment comparisons of child function level (everyday situations: child behavior) at baseline, posttreatment, and 6 months after baseline.

Variable	Baseline^a^ to posttreatment^b^			Baseline to 6-month follow-up^c^			Posttreatment to 6-month follow-up	
	Mean (95% CI)		*P* value	Mean (95% CI)	*P* value	Mean (95% CI)		*P* value
Child behavior total	6.1 (3.0 to 9.2)	<.001		9.4 (5.0 to 13.8)	<.001	3.3 (–0.3 to 6.9)		.07
Transition situations	1.9 (0.6 to 3.1)	.004		3.3 (1.8 to 4.8)	<.001	1.4 (0.2 to 2.7)		.03
Dining situations	1.2 (0.4 to 2.1)	.006		1.8 (0.9 to 2.7)	<.001	0.6 (–0.2 to 1.3)		.12
Situations outside home	1.6 (0.8 to 2.5)	<.001		2.3 (1.0 to 3.6)	.001	0.7 (–0.4 to 1.8)		.20
Home situations	1.3 (0.4 to 2.2)	.005		1.9 (0.7 to 3.2)	.004	0.6 (–0.4 to 1.6)		.25

^a^Measurement before the program started.

^b^Measurement after the program ended.

^c^Measurements 6 months after starting the program.

**Table 8:** Treatment comparisons of parental skills and parental mental health at baseline, posttreatment, and 6 months after baseline.

Variable			Baseline^a^ to posttreatment^b^				Baseline to 6-month follow-up^c^					Posttreatment to 6-month follow-up		
			Mean (95% CI)		*P* value	Mean (95% CI)		*P* value		Mean (95% CI)			*P* value	
**PS^d,e^**														
	Total	N/A^f^		N/A			0.5 (0.4 to 0.7)		<.001		N/A			N/A
	Laxness	N/A		N/A			0.3 (0.1 to 0.6)		.02		N/A			N/A
	Overreactivity	N/A		N/A			0.8 (0.6 to 1.1)		<.001		N/A			N/A
	Hostility	N/A		N/A			0.3 (0.1 to 0.5)		.004		N/A			N/A
**DASS-21^e,g^**														
	Total	N/A		N/A			5.8 (1.4 to 10.3)		.01		N/A			N/A
	Depression	N/A		N/A			1.8 (–0.2 to 3.7)		.07		N/A			N/A
	Anxiety	N/A		N/A			0.1 (–1.6 to 1.7)		.93		N/A			N/A
	Stress	N/A		N/A			3.9 (2.2 to 5.6)		<.001		N/A			N/A

^a^Measurement before the program started.

^b^Measurement after the program ended.

^c^Measurements 6 months after starting the program.

^d^PS: Parenting Scale.

^e^The PS and DASS-21 were measured only at baseline and 6 months after baseline.

^f^N/A: not applicable.

^g^DASS-21: 21-item Depression, Anxiety, and Stress Anxiety Stress Scale.

## Discussion

### Principal Findings

The study showed that the parent training program was effective when it was used in a specialist clinical setting during the COVID-19 pandemic. The program led to significant improvements in children’s externalizing symptoms 6 months after baseline. It improved most of the psychopathology symptom domains we measured, including parent-reported externalizing, internalizing, hyperactivity and peer problems, irritability, and prosocial behavior. The changes in the children’s psychopathology and functioning were fairly similar to the population-based RCT and the child health clinic implementation study [29-32]. It is often assumed that digital interventions are best suitable for those who have minor behavioral problems. However, this study showed that internet-based interventions with telephone coaching are effective for children who have more severe behavioral problems. In the population-based implementation study [32], the mean change for the CBCL total score between baseline and the 6-month follow-up was 15.2 points, while in this study, the mean change was 16.3 points.

The results showed that the program provides parents with feasible parenting skills that they are able to sustain, even after the program ends. The impact that the program had on the children’s social development was remarkable, as the intervention had positive effects on daily transitions and activities, such as getting dressed, behavior when eating, and activities inside and outside the home. The self-reported parenting skills significantly improved, and parents expressed less distress at the 6-month follow up in relation to dealing with their child. This was despite the fact that the intervention was conducted during the COVID-19 pandemic, which was bound to be a stressful time. It is noteworthy that although the effects were maintained at 6 months, according to most of the child psychopathology measures we used, the intervention did not have a long-lasting effect on callous-unemotional traits, which have been associated with poorer treatment outcomes [48].

The number of parents who failed to complete the program was low, and the parents who did were highly satisfied with the program. These findings show that the program was feasible during the height of the COVID-19 pandemic in Finland. One of the keys to successful parent training interventions is the ability to engage and retain parents in the program [49-51]. High dropout rates have been reported by digital interventions, and these have been particularly associated with unguided interventions [50,52-55]. The 12% dropout rate in our study, which included telephone coaching sessions, was much lower than the 30%-50% reported by previous studies on digital parent training interventions [56-60]. There are a number of possible reasons for the low dropout rate, including the fact that the program had a strong background of research-based evidence. The context of the program was well defined, and there were clear inclusion and exclusion criteria. In addition, the parents voluntarily sought help to address their children’s challenges from the family counseling center and the program included weekly telephone coaching on the weekly themes. The program also had a clear structure, and the parents received weekly feedback and support from the family coach. Digital interventions that include guidance and support, such as regular phone calls, have been shown to have a larger effect size on mental health outcomes than smartphone interventions without any personal support [61].

### Comparison With Previous Works

Even though there has been a lot of research published about parent training, none of this has addressed how an internet-based parent training program was implemented during exceptional circumstances, such as the COVID-19 pandemic. It was possible to implement the program during the pandemic because it did not require face-to-face meetings and the parents were not required to leave home. The findings of this study are also relevant for other types of crises, where the providing face-face services is not feasible.

### Strengths and Limitations

The strengths of the study were that the SFSW is an established program that has already been the subject of an RCT and has been successfully implemented in primary care child health clinics in Finland [29-32]. The study was carried out at a time of international crisis, during the height of the pandemic in Finland, which meant that it was tested during stressful and rigorous social distancing conditions. Despite this, it had a good retention rate, high parental satisfaction, and engagement. The 6-month follow-up assessment provided good data on how feasible and sustainable the program was.

Some limitations should also be noted. First, the COVID-19 pandemic meant that treatment and family counseling services could not be provided in the usual way, and this meant that it was not possible and ethical to conduct the study as an RCT. The study design did not make it possible to draw direct conclusions about the effectiveness of the parent training program, because the study did not have an intervention-control group design, but parental satisfaction was positive. However, in previous studies, we have been able to show the long-term effectiveness of the program. In the RCT intervention group, the changes in children’s conduct problems and parents’ parenting skills were maintained at the 2-year follow-up [29,30]. In addition, we compared a large implementation sample with the RCT sample [32]. The RCT intervention group and the implementation group did not differ at the 6-month follow-up. This means that the program was effective and may have benefits over traditional group-based treatment approaches when the goal is to identify children at risk in the community at an early stage.

Another limitation was that only parental reports of child behavior were used in the analyses. Direct observations of parenting, and clinical observations or teacher ratings, would have helped validate the reported changes, but social distancing, including school closure, meant this was not possible. This also made it impossible to obtain pretest and posttest data, for example, from teachers. The study also covered children aged 3-8 years, so self-reports were not really feasible. Finally, the participants were limited to those who could speak, read, and write Finnish or Swedish and had access to a computer or smartphone.

### Conclusion

The internet-based parent training program with telephone coaching (SFSW) was successful in helping parents tackle child behavioral problems in children aged 3-8 years. The participants reported significant improvements in parenting skills and child psychopathology and functioning. Satisfaction was high, and dropout rates were low. These findings are remarkable because the study was conducted during the COVID-19 pandemic, when health care services and schools were in lockdown and parents were told to work at home if they could.

Providing sustainable key services during crises is a major challenge for society. Social distancing during the height of the pandemic meant that the face-to-face services that have traditionally proved successful in addressing disruptive child behavior were simply not possible. The COVID-19 pandemic has highlighted the importance of exploring remote, digital, or digitally assisted solutions for ensuring that young children, and their families, are provided with prompt support for mental health problems. This study demonstrated that technology can provide effective alternatives to traditional face-to-face interventions and can overcome a number of barriers during crises. Technology can be used to provide the right treatment at the right time, with high levels of support and fidelity, greater access, convenience, and reduced costs and time.

## Appendix

Multimedia Appendix 1 Schedule of questionnaires filled by parents during the program.

Multimedia Appendix 2 Satisfaction-related questions in the parent training program.

## Abbreviations

ARI: Affective Reactivity Index
CBCL: Child Behavior Checklist-Parent Report Form
DASS-21: 21-item Depression, Anxiety, and Stress Scale
ICU: Inventory of Callous-Unemotional Traits
PS: Parenting Scale
RCT: randomized controlled trail
SDQ: Strengths and Difficulties Questionnaire
SFSW: Strongest Families Smart Website
