# Supplementary material for: An Internet-Based Parent Training With Telephone Coaching on Managing Disruptive Behavior in Children at Special Family Counseling Centers During the COVID-19 Pandemic: Feasibility Study
Source: JMIR Pediatr Parent. 2022 Nov 2;5(4):e40614. doi: 10.2196/40614 (PMC9635457; doi:10.2196/40614)
Supplement: Multimedia Appendix 1 [file pediatrics_v5i4e40614_app1.docx]

Table S1. Schedule of questionnaires filled by parents during the program

| **Questionnaire** | | Baseline^a^ | Posttreatment^b^ | After 6 months^c^ |
| --- | --- | --- | --- | --- |
|  | SDQ^f^ | x^d^ | x | x |
|  | ARI^g^ | x | x | x |
|  | CBCL/1.5-5^h^ | x | -^e^ | x |
|  | ICU^i^ | x | - | x |
|  | Everyday situations (child behavior) | x | x | x |
|  | Parenting scale | x | - | x |
|  | DASS-21^j^ | x | - | x |
|  | Satisfaction | - | x | - |

^a^Measurement before the program started
^b^Measurement after the program ended
^c^Measurements 6 months after starting the program
^d^Conducted

^e^Not applicable
^f^SDQ = Strengths and Difficulties Questionnaire
^g^ARI= questionnaire for irritability
^h^CBCL/1.5-5= Child behavior checklist for preschool children
^i^ICU= Inventory of Callous-Unemotional Traits
^j^DASS-21= 21-item Depression, Anxiety, and Stress Scale.
