# Supplementary material for: An Internet-Based Parent Training With Telephone Coaching on Managing Disruptive Behavior in Children at Special Family Counseling Centers During the COVID-19 Pandemic: Feasibility Study
Source: JMIR Pediatr Parent. 2022 Nov 2;5(4):e40614. doi: 10.2196/40614 (PMC9635457; doi:10.2196/40614)
Supplement: Multimedia Appendix 2 [file pediatrics_v5i4e40614_app2.docx]

**Table S2.** Satisfaction-related questions in the parent training program (n=45) excluding 5 parents who didn´t answer the satisfaction questionnaire.

| **Satisfaction-related factors ^a^** | | | Parents, n (%) |
| --- | --- | --- | --- |
| **Overall impact: parent training program** | | | |
|  | ***The program matched my expectations*** | | |
|  |  | Disagree/neutral | 6 (13.3) |
|  |  | Agree | 39 (86.7) |
|  |  | ***The program met my needs*** |  |
|  |  | Disagree/neutral | 8 (17.8) |
|  |  | Agree | 37 (82.2) |
|  | ***I would recommend the program to my friends, if they were in need of similar help*** | | |
|  |  | Disagree/neutral | 1 (2.2) |
|  |  | Agree | 44 (97.8) |
|  | ***In case I would need help in the future, I would enroll to the program again*** | | |
|  |  | Disagree/neutral | 11 (24.4) |
|  |  | Agree | 34 (75.6) |
|  | ***Generally speaking, how content have you been with the Strongest Family program?*** | | |
|  |  | Disagree/neutral | 3 (6.7) |
|  |  | Agree | 42 (93.3) |
| **Overall impact: effects on parenting skills** | | | |
|  | ***I have learned skills, which have been helpful to me as a parent*** | | |
|  |  | Disagree/neutral | 0 (0.0) |
|  |  | Agree | 45 (100.0) |
|  | ***My ability to act as a parent has developed*** | | |
|  |  | Disagree/neutral | 1 (2.2) |
|  |  | Agree | 44 (97.8) |
|  | ***I trust more in my abilities to act as a parent*** | | |
|  |  | Disagree/neutral | 6 (13.3) |
|  |  | Agree | 39 (86.7) |
|  | ***My relationship with my child has improved*** | | |
|  |  | Disagree/neutral | 12 (26.7) |
|  |  | Agree | 33 (73.3) |
|  | ***My stress levels have been relieved*** | | |
|  |  | Disagree/neutral | 18 (40.0) |
|  |  | Agree | 27 (60.0) |
| **Direct impact: family coach** | | | |
|  | ***The coach respected my views on parenting*** | | |
|  |  | Disagree/neutral | 0 (0.0) |
|  |  | Agree | 45 (100.0) |
|  | ***The coach was professional*** | | |
|  |  | Disagree/neutral | 1 (2.2) |
|  |  | Agree | 44 (97.8) |
|  | ***The coach encouraged problem solving*** | | |
|  |  | Disagree/neutral | 2 (4.4) |
|  |  | Agree | 43 (95.6) |
|  | ***I could form a successful working relationship with the coach*** | | |
|  |  | Disagree/neutral | 3 (6.7) |
|  |  | Agree | 42 (93.3) |

^a^Disagree/neutral combines strongly disagree, disagree and not agree or disagree. Agree combines agree and strongly agree.
